# The Role of Family Factors in the Development of Dental Anxiety in Children

**DOI:** 10.3390/medicina60010180

**Published:** 2024-01-19

**Authors:** Dorotea Petrović, Odri Cicvarić, Marija Šimunović-Erpušina, Nataša Ivančić Jokić, Danko Bakarčić, Petra Bučević Sojčić, Hrvoje Jurić

**Affiliations:** 1 Department of Pediatric Dentistry, Faculty of Dental Medicine, University of Rijeka, 51000 Rijeka, Croatia; dorotea.petrovic@uniri.hr (D.P.); marija.simunovic.e@gmail.com (M.Š.-E.); natasa.ivancic.jokic@fdmri.uniri.hr (N.I.J.); danko.bakarcic@fdmri.uniri.hr (D.B.); 2 Dental Clinic, Clinical Hospital Center Rijeka, 51000 Rijeka, Croatia; 3 Department of Pediatric and Preventive Dentistry, School of Dental Medicine, University of Zagreb, 10000 Zagreb, Croatia; pbucevic@sfzg.hr; 4 University Dental Clinic, University Hospital Centre Zagreb, 10000 Zagreb, Croatia

**Keywords:** dental anxiety, children, family factors, parents, socio-economic factors

## Abstract

*Background and Objectives*: In the literature, the influence of parents who suffer from dental anxiety and a previous unpleasant experience at the dentist are cited as the two most common causes of dental anxiety in children. The aim of this study is to investigate the relationship between socioeconomic factors and the development of dental anxiety in children aged 9 to 12 years. *Materials and Methods*: A total of 131 children and their accompanying parents/guardians participated in the cross-sectional study. The children were divided into an experimental group, which visited a specialised office for paediatric and preventive dentistry for the examination, and a control group, which visited a primary care dental office. During the visit, the children completed questionnaires on dental anxiety (CFSS-DS). Parents completed a socioeconomic questionnaire and a dental anxiety questionnaire for adults (CDAS). *Results*: The results showed a statistically significant positive predictor: parental dental anxiety as measured by the CDAS. In addition, the *t*-test showed that children who visited a specialised dental office did not show a statistically significant increase in dental anxiety compared to children who visited a primary care dental office. *Conclusions*: With this study, we confirm the influence of parental dental anxiety on the development of dental anxiety in children. The socioeconomic status of the family and the type of dental office do not play a statistically significant role in the development of dental anxiety in children.

## 1. Introduction

Dental anxiety (DA) is defined as a strong, negative feeling associated with a dental procedure, regardless of the general condition, preceded by a negative dental experience [1,2]. Dental anxiety is a universal phenomenon that affects patients regardless of their age and negatively influences the quality of life related to oral health in children and adults [3]. The patient’s experience is subjective and can vary greatly depending on the person’s physical and mental condition [4]. Sometimes, a child may express their fears and anxieties, while others may express them through behaviors such as crying, fussing, interrupting conversations or play, and even trying to run away from the caregiver. It can also be accompanied by significant physical changes, such as an increase in heart rate, other hemodynamic changes and the release of stress hormones [5].

The main effects of dental fear are deterioration in oral health and the perpetuation of a vicious cycle of avoiding or canceling already scheduled appointments. Dental appointments are only attended when pain or major discomfort occurs, which further increases the feeling of anxiety [6]. Fear of the dentist is one of the main causes of irregular visits to the dental office, along with a lack of time, financial resources and accessibility of the office staff [6]. The prevalence of dental anxiety in children varies between 5 and 33% depending on the study [1]. The causes of dental anxiety can be divided into several categories: biological causes (individual temperament, genetics and epigenetics) [7], social causes (parental dental anxiety, family socioeconomic status (SES), parental adjustment before dental procedures and parental expectations of children’s behavior in the dental environment) and factors of the dental environment related to the appearance of the dental office, treatment and surroundings [8]. There are currently no well-defined treatment or control options for dental anxiety. Further research is needed to make treatment more accessible and to provide dental procedures adapted to patients with dental anxiety through additional education of dentists [9].

Children behave differently during dental procedures. Therefore, a dentist must know the characteristics of the child’s psychological development, which depend on the child’s age, so that he can respond to each patient individually [10]. Managing children’s behavior during dental treatment is extremely important. Poorly controlled pain affects the child’s well-being and may lead to a decreased ability to effectively manage future pain [11]. Dental anxiety can be controlled by psychotherapeutic interventions, pharmacological interventions, or a combination of both, depending on the dentist’s expertise and experience, the degree of dental anxiety, the patient’s characteristics and the clinical situation [12]. Dental anxiety can vary from very mild to extreme and interacts with the urgency of treatment, so different approaches to reducing anxiety may be appropriate depending on the level of anxiety [13]. Some treatment methods can reduce anxiety. With an appropriate approach adapted to the age of the child or patient and a detailed explanation of the procedure, we can influence the reduction in anxiety. Patients may show less anxiety for treatments that do not require anaesthesia such as minor anterior restorations [14] or bleaching procedures [15]. A good relationship between the patient and dentist is key to managing anxiety.

Several factors influence a child’s oral health, from the child’s behavior to family and society. Parental variables that are directly related to children’s oral health include sociodemographic characteristics, oral health behaviors, knowledge, fears, and many others. Since the family environment influences the children’s oral habits, it is necessary to assess not only the child’s oral status, the presence of carious lesions and concomitant clinical situations in the child, but also related family factors [16]. Goettems et al. [17], who investigated the relationship between the influence of maternal dental anxiety, the pattern of dental check-up utilization and the child’s perception of quality of life, found that children in Brazil from families with a higher socioeconomic status were five times more likely to have used dental health services than children with a lower economic status. Previous studies have suggested that socioeconomic status may be a key factor in dental anxiety, with children from the lowest socioeconomic groups showing higher levels of dental anxiety [18]. This suggests that socioeconomic status may be a key factor in dental anxiety. However, this was not observed in the results of Amorim et al. [18]. Ramseier et al. [19] investigated the relationship between socioeconomic factors and dental anxiety in a group of 2240 participants aged 43.5 ± 16.0 years and found a statistically significant inverse relationship: a higher level of education correlated with a lower level of dental anxiety. The results emphasise the potential role of education in coping with dental anxiety and its impact on dental health and well-being [19].

Back in 2001, Majstorovic et al. [20] showed that early negative medical experiences were the main factor for the development of dental fear and that maternal dental anxiety and socioeconomic circumstances were less important factors in children aged 5.5 to 12.5 years in Croatia. The study by Škrinjarić et al. [21] showed that maternal dental anxiety was significantly associated with the child’s dental anxiety, but not with the child’s dental health. The most recent study by Šimunović et al. [22], which was conducted in six European countries, including Croatia, also showed a statistically significant correlation between the parents’ dental anxiety and that of the children. Research into the influence of socioeconomic status in Croatia on the development of dental anxiety in children has been insufficient.

The aim of this study was to investigate the relationship between family factors and the development of dental anxiety in children aged 9 to 12 years. This study investigated whether socioeconomic status or other parental factors could have a predictive value for dental anxiety. Knowing the predictors that may trigger dental anxiety could be very helpful for dentists in the behavioral management of their patients. Therefore, these findings could provide guidance for better promotion of oral health and thus quality of life.

The null hypothesis of this study is that the child’s dental anxiety is related to the socioeconomic status of the family and parents’ dental anxiety.

## 2. Materials and Methods

### 2.1. Study Design

The study was conducted as a cross-sectional study [23] and included the clinical population of children and their parents in Croatia in the Osijek-Baranja County region. The study was approved by the Ethics Committee of the University of Zagreb, School of Dental Medicine (No. 05-PA-4-7-XI-1/2020, on 20 October 2022) and the Ethics Committee of the Health Center of the Osijek-Baranja County (No. 03-3160-1122, on 2 December 2022). Written informed consent was obtained from all parents prior to participation in the study.

### 2.2. Sample

Children of both sexes participated in the study. In line with previous studies on parental roles, parents of both sexes were included in the study. The study was conducted in the dental offices of the Osijek-Baranja County Health Centre. The sample size was determined based on the calculation of the statistical power of the test of 80% in the programme G*Power 3.1.9.7. at a significance level of α = 0.05. Based on the number of predictors, the minimum number of respondents was 118. Data were collected for 131 children. In total, 101 children came to a specialist in paediatric and preventive dentistry, and the data of 30 children who came to a primary care dentist were also collected as a control group. The group of children studied was between 9 and 12 years old. The children completed the questionnaires that were presented in paper form independently, before being examined by a dentist under the supervision of the researcher. The parents completed the questionnaires independently and under the supervision of the researcher. The clinical examinations were carried out by selected dentists and specialists in paediatric and preventive dentistry to prevent the first encounter with a new dentist and/or a new dental team leading to increased dental anxiety in children.

The inclusion criteria were as follows: children aged 9–12 years, children who consented to participate in the study, i.e., parents who consented to the child’s participation, and children with previous dental experience. The exclusion criteria were as follows: children outside the age limit, children suffering from diseases that prevent independent communication with the dentist, children who come to the dental office for emergencies, and painful and swollen children. 

### 2.3. Research Instruments

The questionnaires were adapted to the age of the children aged 9 to 12 and to the parents, validated and translated into Croatian. In order to assess the SES of the respondents, parents were asked to describe their family’s material circumstances as well as their parents’ level of education and housing conditions. The Corah Dental Anxiety Questionnaire (CDAS) and the Children’s Fear Survey Schedule-Dental Subscale (CFSS-DS) were used for the questionnaires. The reliability calculated as Cronbach’s α for the CDAS in our sample was 0.92 and for the CFSS-DS 0.84. The Children’s Fear Survey Schedule-Dental Subscale (CFSS-DS) is a validated psychometric instrument for assessing dental fear in children. It consists of 15 questions relating to different aspects of dental situations. The statements are rated on a five-point Likert scale (from 1—I am not afraid at all to 5—I am very afraid). The possible scores range from 15 to 75, with higher scores indicating increased dental anxiety. Previous studies have shown that scores below 32 are not clinically significant. The Corah’s Dental Anxiety Questionnaire (CDAS) is a validated psychometric instrument for measuring dental anxiety and fear in adults. Anxiety and fear of dental procedures is measured using four different dental situations on a five-point scale. The total possible score is 20, with scores below 9 indicating mild anxiety and scores between 15 and 20 indicating a high level of anxiety (phobia). 

### 2.4. Statistical Analysis

The statistical software programme IBM SPSS Statistics 23.0 (IBM Corp, Armonk, NY, USA; https://www.ibm.com/spss (accessed on 17 January 2024); 2015) and the SPSS module PROCESS+3.0 [24] were used for the mediation analysis. The following statistical methods were used to process the results: descriptive data, correlation estimation (Pearson, Spearman), multiple regression analysis and mediation analysis. The descriptive statistics method was used to determine the basic statistical parameters (means, standard deviations). Pearson’s correlation coefficient was calculated to determine the relationship between specific pairs of variables. The multiple regression method was used to determine the relationship between each predictor variable and children’s dental anxiety as a criterion. Using multiple regression, we were able to show how much each of the predictors separately contributed to explaining the variance of the criteria. The included predictors, variables with which we want to explain the criterion, are SES and CDAS (parents’ dental anxiety) and the type of dentist the children visit, i.e., a specialist or general dentist. To test the hypothesis of this study, family socioeconomic status was chosen as the independent variable, while the dependent variable is the child’s dental anxiety. The mediating variable is the parents’ dental anxiety. The independent variable may influence the dependent variable directly or through an indirect effect.

## 3. Results

### Descriptive Statistics

Of 131 participants, 69 (52.7%) were male. The mean age of the children was 10.5 years (SD = 1.17 years). The parents in the sample were predominantly mothers (*N* = 98, 74.8%), most of whom had a vocational secondary school degree (*N* = 79, 60.3%) or a university degree (*N* = 29, 22.1%). The majority of parents are permanently employed (*N* = 99, 75.6%), a smaller number are unemployed (*N* = 18, 13.7%), and the rest are retired, occasionally employed or caring parents. The vast majority of parents in the sample are married or cohabiting (*N* = 118, 90.1%) and live in their own apartment or house (*N* = 124, 94.7%). In order to assess the SES of the respondents, the parents were asked to describe the material circumstances of their family. The results are shown in Table 1.

The descriptive data of the research sample are shown in Table 2.

Before further analyses were carried out, the normality of the distributions of the variables mentioned was tested. To determine the deviation from normality, the Shapiro–Wilk test was used, the results of which are shown in Table 2. The distribution of most variables statistically significantly deviated from normality, so we also presented the indices of asymmetry and flattening of the distributions.

In addition to the sample of children who visited a paediatric and preventive dentistry specialist, we also collected a control sample of children who visited a primary care dentist (DDM) (*N* = 30). To determine how the differences between these two samples manifest themselves, we compared their scores on measures of dental health and psychological measures. The comparisons were made using the *t*-test for independent samples. This test determines the significance of the difference between the arithmetic means of two groups in relation to the common standard deviation. The results are shown in Table 3.

For certain measures used in this study, it is possible to divide the results obtained into different groups. For the CFSS-DS, a score of 38 or higher is considered anxiety. For the CDAS, a score of 9 or less is considered normal, 9 to 12 is considered moderate, 13 to 14 is considered high, and 15 or more is considered phobic. Table 4 and Table 5 show the distribution of results by category, which are also subdivided according to whether the child sees a primary care dentist or a paediatric and preventive dentistry specialist. The percentages in the table can be used for a rough comparison between the groups.

To determine the relationship between the variables included in our analysis, we calculated Pearson’s correlation coefficients between all pairs of variables. The correlation matrix of the results is shown in Table 6. Statistically significant correlations are shown in bold. A moderate positive correlation was found between the CFSS-DS and CDAS scores (r = 0.29; *p* < 0.01).

**Figure 1:**
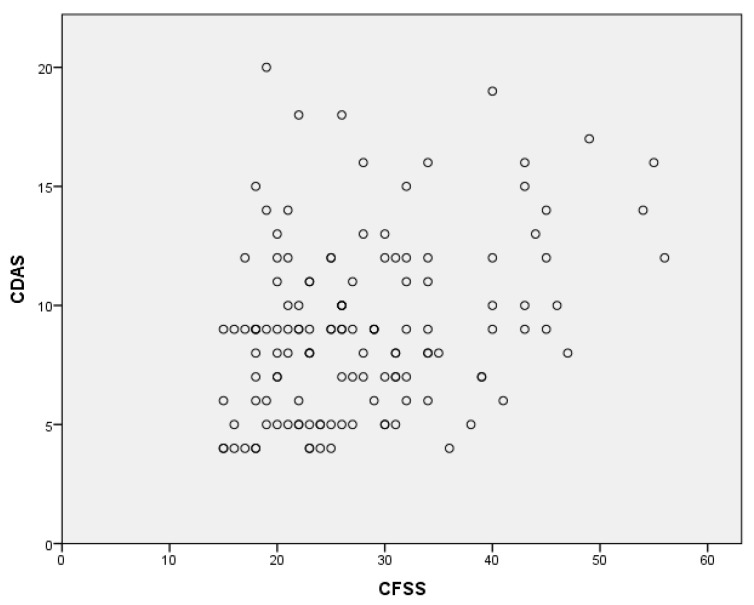
Scatterplot of the relationship between the CFSS and CDAS values.

A graphical representation of the positive correlation between the parent’ dental anxiety and the child’s dental anxiety is shown in Figure 1.

In our study, we wanted to explain what children’s dental anxiety (measured with the CFSS-DS) is related to. To answer this question, we conducted a multiple regression analysis with children’s dental anxiety as a criterion and demographic predictors. The results are shown in Table 7.

**Figure 2:**
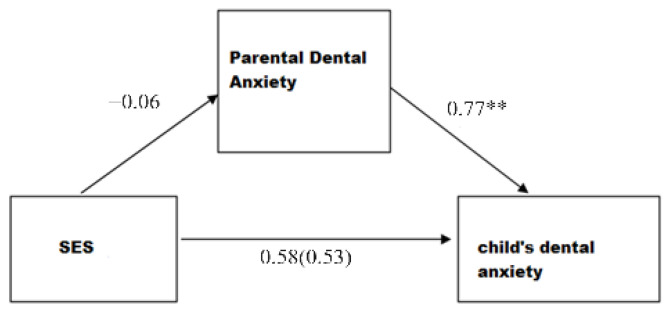
Graphical representation of the mediation model of the relationship between SES and the child’s dental fear, with parental dental fear as the mediator (** *p* < 0.01).

An additional statistical analysis was conducted to further investigate the nature of the relationship between socioeconomic status and dental anxiety in children. A mediation model was created with SES as a predictor, parental dental anxiety as a mediator and child dental anxiety as a criterion. This mediation model is shown in Figure 2 together with the unstandardized correlation coefficients of the included variables.

No statistically significant relationship was found between SES and children’s dental anxiety, either before or after, including parental dental anxiety as a mediator (*p* > 0.05). The results of our sample show that SES cannot be directly or indirectly (via parental dental anxiety) associated with children’s dental anxiety.

## 4. Discussion

Dental anxiety is a common phenomenon in children who come to the dentist’s office. Severe dental anxiety in children who visit the dental office not only leads to the failure of normal dental treatment, but also casts a psychological shadow on dental anxiety in adult patients [25]. Assessment of dental anxiety using a questionnaire is the most commonly used method for assessing dental anxiety in pediatric patients. Since children, especially depending on the age of the child, cannot always clearly express potential dental anxiety and almost always come accompanied by their parents, questionnaires for self-assessment of dental anxiety are often used today, which the child completes independently, but parents also assess the potential anxiety felt by the child in special questionnaires [25]. The results of this study show that the mean score of the CFSS-DS questionnaire completed by children visiting a specialist dental practice is 27.76 (SD = 8.97). This means that in the studied group of children, more than 86% of them experience some level of fear of dental procedures but do not report dental anxiety, while according to the thresholds for classification, slightly more than 13% of them have an abnormal level of dental anxiety. In their study, Wu and Gao [1] found that 33.1% of children who completed the CFSS-DS showed dental anxiety. In total, 15.3% of the children who completed the questionnaire showed a moderate level of dental anxiety by achieving numerical scores between 32 and 39 on the questionnaire, while 17.8% of the children who completed the questionnaire achieved scores above 39, placing them in the category of children with extreme dental anxiety. Alsadat et al. [26], who investigated the relationship between children’s dental anxiety and dental caries, reported an average CFSS-DS test score of 26.09, with only 12.50% of children reporting higher levels of dental anxiety. Differences in research results may depend primarily on cultural differences, the availability of dental care and the way a child is brought up.

Based on the research findings, we conclude that sociodemographic factors, i.e., the child’s gender and self-rated SES, as well as the dentist the child visits, whether a specialist or a primary care doctor, are not statistically significant predictors of dental anxiety in children. Shindova et al. [27] found in their study that gender has an influence on dental anxiety. Female respondents to the CFSS-DS questionnaire reported a higher level of dental anxiety than male respondents. The study by Majstorović et al. [28] found that dental anxiety is more common in adolescent girls than in boys. In a study of a population of children aged 6 to 12 years, cross-cultural studies on the aetiology of dental anxiety could help to clarify these contradictory findings regarding the relationship between gender and dental anxiety [29]. At the bivariate correlation level, we found a statistically significant association between female gender and dental anxiety in children, but in the regression analysis, the child’s gender did not prove to be a statistically significant predictor.

Some authors found that the level of anxiety decreases with increasing age [30]. Boka et al. [31] found no statistically significant correlation between age and dental anxiety. In the study by Lima et al. [32] with children aged 7 to 9 years, they concluded that the age of the child influenced the level of dental anxiety and emphasised that younger children have a higher level of dental anxiety. They cite the acquired ability to deal with situations that we have previously experienced as the reason for the decrease in dental anxiety. Alshoraim et al. [33] concluded in their study that age cannot be a reliable predictor of dental anxiety in children because oral status and the influence of cultural differences significantly affect the child’s age or development and thus dental anxiety, whereas in this study we did not use age, as a predictor of dental anxiety because the age range of the children who participated in the study was very small. Uzel et al. [34], who investigated the influence of risk factors on the development of dental anxiety, did not find increased dental anxiety in children of lower socioeconomic status. In the study by Yildirim [35] on the adult population of periodontal patients in Turkey, a correlation was found between lower socioeconomic status and increased dental anxiety in patients. Muneer et al. [36] investigated dental anxiety and the factors influencing it in adults and came to the conclusion that people with a lower socioeconomic status suffer more frequently from dental anxiety. In fact, socioeconomic status was a parameter for a number of behavioural, social, economic, and psychological covariates [35]. In many countries, the utilisation of dental services is related to the availability of health care, i.e., socioeconomic status. In the Republic of Croatia, health care, including dental care, is free for children up to the age of 18, while adults pay for or have free access to health care depending on their employment status. According to Uziel et al. [37], parents’ education level is a significant predictor of a child’s dental anxiety, with a focus on the mother’s education level, although they also mention the influence of the father’s education as a factor. The largest proportion (60.31%) of parents in our study had only completed high school. University education was reported by 22.14% of parents. On the other hand, Amorim et al. [16] did not find increased dental anxiety in children as a function of parental educational status and note that culture and parental anxiety may influence the results of other studies that suggest a relationship between the aforementioned factors, such as the study by Rantavuori and coworkers [38].

Although age, gender, temperament and development play a particular role in regulating children’s stress responses, perhaps the most influential factor is experience with the parents’ stress response model [39]. It is known that children who witness their parents’ anxiety are likely to acquire such a view and consequently have painful experiences at a young age, which is a strong factor in the development of dental anxiety [40]. On the other hand, based on their research findings, Wu et al. [1] suggest that the child’s dental anxiety is not directly related to the parent’s dental anxiety, but is influenced by the child’s family structure and siblings, which play an important role in the development of the child’s dental anxiety. Ćorić et al. [41] observe a statistically significant correlation between the mother’s dental anxiety and the occurrence of dental anxiety in children, although they also note the influence of the father on the child, albeit to a lesser extent. Mothers are often more involved in childcare, which could explain this phenomenon. However, due to the change in gender roles, fathers and mothers today have the same influence on child rearing. Psychological knowledge about fear in the form of an emotion that can be learned or acquired by observing the environment makes parents role models whose behaviour and expression of emotions the child tries to copy. Avoiding dental procedures, expressing fear of dental procedures, pain, injections, irregular oral hygiene and eating habits can be learned by the child from the parents or guardians [41]. When conducting our study, a moderately positive correlation was found between the results of the CFSS-DS and the CDAS, suggesting that parents who are more likely to be dentally anxious are also more likely to be dentally anxious than their children. 

With regard to the child’s behaviour and the need for dental examinations and interventions, based on the results of our study, we found no statistically significant differences in the child’s and parents’ dental anxiety between the experimental and control groups. Based on the literature reviewed, we expected that children’s dental anxiety would be higher in the group visiting a paediatric and preventive dentistry specialist. Based on our results, it appears that children are equally anxious about dentistry, even if the need to see a paediatric and preventive dentistry specialist was not identified. Krikken et al. [42] investigated the possible relationship between children’s dental anxiety and the referral of a child to a specialty dental practise compared to a primary care dental practise and observed an increased level of dental anxiety in the group of children referred to a specialty practise. Children were most often referred to a specialty clinic for behavioural problems. The referral of a child to a specialist depends in part on the interaction between the child and the dentist and the parents. In this study, we found that there is no difference in the anxiety of children who come to paediatric and preventive dentistry specialists compared to primary care dentists. This suggests that the severity of the procedure, the dental problem or the dental environment, which differ depending on the type of procedure, have no influence on the development of dental anxiety in children. On the other hand, psychological variables and parental dental anxiety are related to the presence of dental anxiety in children. The child’s experience of visiting the dentist’s office, the child’s preparation at home for the visit or the performance of the dental procedure are more likely to be related to dental anxiety. Continuous visits to the dentist with the appearance of the first deciduous teeth in the jaw, as well as familiarization with dental procedures through games or virtual content, with the encouragement of the child and visits where the child has the opportunity to see the parent in a situation where a preventive dental examination or a necessary dental procedure is performed, are certainly recommendations that can influence the child’s later perception of the dental environment. Visits to the dental office that were not preceded by pain or discomfort related to the condition of the oral cavity, as well as the parents’ positive attitude towards dental care, can influence or contribute to reducing the child’s attitude and anxiety.

The conducted research has certain limitations. Since this research is a cross-sectional study, based on the results obtained, we can talk about the possible relationship of certain variables, but not about the direct influence of different factors on the development of dental anxiety in children. Given the dental environment and the presence of parents in the dental office as well as the examiner as a new person in the dental environment, children may show a lower level of dental anxiety to show their age-related courage and maturity. In this study, only one of the child’s parents completed the questionnaire. Therefore, we have information about his dental anxiety, but not about the anxiety of the other parent. Of course, it should be taken into account that the results of the above-mentioned questionnaire may be different for both parents. 

The factors influencing dental anxiety and its negative effects in society need to be researched more comprehensively in order to obtain balanced data representing a sample of the entire population [36]. Based on the clear and credible evidence thus collected on the influence of certain etiological factors, it would be possible to reduce the incidence of dental anxiety from an early age by educating parents and dentists with appropriate preventive measures. This will certainly improve oral health, which will have a direct impact on the quality of life of each individual. Furthermore, by identifying the key factors for the development of dental anxiety in children, it will be possible to create clear guidelines and reliable tools to assess and prevent the development of dental anxiety regardless of the child’s age, which will ultimately lead to better care for patients throughout their lives. Future studies should not only include a larger number of respondents, but also be conducted in a larger area and take into account the cooperation of several dental faculties, including the population of neighboring countries with approximately similar oral hygiene habits and similar incidence of dental caries, such as the study conducted by Šimunović et al. [22] that includes the target population of children aged 9 to 12 years, a crucial period just before the onset of puberty. Also, future research should include parents of both sexes, an equal number of respondents in all groups and the influence of siblings on the development of dental anxiety.

## 5. Conclusions

The influence of the parents on the child, whether positive or negative, is reflected in the child’s behavior, but also in their perception of the environment. The results obtained indicate a statistically significant correlation between children’s dental anxiety and that of their parents. Further studies involving both parents would provide more comprehensive results. The influence of the environment in which the child grows up and the socioeconomic status of the family have been shown in some studies to be important factors in the development of dental anxiety, but no correlation between these factors was found in the current study. Educating the parents about the child’s approach and acclimatizing the child to the dental procedures and the new environment will influence the child’s perception of the dental environment and reduce any dental anxiety that may be present.

## Figures and Tables

**Table 1:** Self-assessment of the respondents’ material circumstances.

	Frequency	Percentage %
Poorly	2	1.53
Moderately	25	19.08
Good	84	64.12
Very good	20	15.27
Total	131	100.0

**Table 2:** Descriptive data of the sample (*N* = 131).

Variable	*N*	*M*	*SD*	Minimum	Maximum	*S-W*	*p*(*S-W*)	Skewness	Flattening
Dental anxiety of a child (CFSS-DS)	131	27.79	9.26	15.00	56.00	0.93	<0.01	0.95	0.43
Dental anxiety of a child (CFSS-DS)	131	9.04	3.64	4.00	20.00	0.94	<0.01	0.74	0.20

*N* = number of participants for whom data were collected; *M* = arithmetic mean, *SD* = standard deviation; *S*-*W* = Shapiro–Wilk test of normality of distribution; *p*(*S*-*W*) = significance level of the Shapiro–Wilk test.

**Table 3:** Comparison of research and control samples (*N* = 131).

	Type of Doctor	*N*	*M*	*t*	*df*	*p*
Dental anxiety of a child (CFSS-DS)	DDM	30	27.90	0.07	129	0.94
	specialist	101	27.76			
Dental anxiety of a parent (CDAS)	DDM	30	8.60	−0.75	129	0.45
	specialist	101	9.17			

*N* = number of participants for whom data were collected; *M* = arithmetic mean, *t* = value of *t*-test; *df* = degree of freedom, *p* = significance level.

**Table 4:** Classification of results for CFSS-DS. (*N* = 131).

		CFSS Category		Total
		Normal	Abnormal	
DDM	*N*	22	8	30
	%	73.33	26.67	100.00
Specialist	*N*	87	14	101
	%	86.14	13.86	100.00
Total	*N*	109	22	131
	%	83.21	16.79	100.00

**Table 5:** Classification of results for CDAS. (*N* = 131).

		CDAS Category				Total
		Normal	Midle	Moderate	Severe Anxiety (Phobia)	
DDM	*N*	17	8	1	4	30
	%	56.67	26.67	3.33	13.33	100.00
Specialist	*N*	43	43	7	8	101
	%	42.57	42.57	6.93	7.92	100.00
Total	*N*	60	51	8	12	131
	%	45.80	38.93	6.11	9.16	100.00

**Table 6:** Intercorrelations of the tested variables. (*N* = 131).

	Type of DDM	SES	CDAS	CFSS
Children gender	0.04	0.01	0.10	0.09
Type of DDM		−0.09	0.07	−0.01
SES			−0.03	0.06
CDAS				0.33 **
CFSS				

The child’s gender is coded so that boys are given a score of 0 and girls are given a score of 1. We coded the type of dentist (specialist in paediatric and preventive dentistry and specialist in primary care dentistry) so that children who come to a specialist in primary care dentistry score 0 and children who come to a specialist in paediatric and preventive dentistry score 1. ** *p* < 0.01.

**Table 7:** Results of multiple linear regression with children’s dental anxiety as the criterion (*N* = 131).

Predictor	Beta	*p*
Children gender	0.06	0.51
Type of DDM	−0.02	0.77
SES	0.06	0.46
Dental anxiety of parents (CDAS)	**0.32**	<0.01
*F*	4.06	
*df*	4/126	
*p*	<0.01	
*R* ^2^	0.11	

We coded the type of dentist (specialist in paediatric and preventive dentistry and specialist in primary care dentistry) so that children who come to a specialist in primary care dentistry score 0 and children who come to a specialist in paediatric and preventive dentistry score 1. Beta = the magnitude of the beta coefficient of a single predictor; *F* = F-quotient of the regression analysis model; *df* = degrees of freedom; *p* = significance level of the predictor, i.e., the regression analysis model; *R*^2^ = percentage of criterion variance explained by the predictors in the model. A statistically significant positive predictor was identified: parents’ dental anxiety, measured with the CDAS.

## Data Availability

All the data used in this study are available on request from the corresponding author.
